# Ultrasonography and color Doppler in juvenile idiopathic arthritis: diagnosis and follow-up of ultrasound-guided steroid injection in the wrist region. A descriptive interventional study

**DOI:** 10.1186/1546-0096-10-11

**Published:** 2012-04-21

**Authors:** Louise Laurell, Michel Court-Payen, Susan Nielsen, Marek Zak, Anders Fasth

**Affiliations:** 1 Department of Pediatrics, Skåne University Hospital, Lund University, Lund, Sweden; 2 Department of Diagnostic Imaging, Gildhøj Private Hospital, University of Copenhagen, Copenhagen, Denmark; 3 Department of Pediatrics, Rigshospital, University of Copenhagen, Copenhagen, Denmark; 4 Department of Pediatrics, Rigshospital, University of Copenhagen, Copenhagen, Denmark; 5 Department of Pediatrics, University of Gothenburg, Gothenburg, Sweden

**Keywords:** Ultrasonography, Color Doppler, Juvenile idiopathic arthritis, US-guided steroid injection, Wrist

## Abstract

**Background:**

The wrist region is one of the most complex joints of the human body. It is prone to deformity and functional impairment in juvenile idiopathic arthritis (JIA), and is difficult to examine clinically. The aim of this study was to evaluate the role of ultrasonography (US) with Doppler in diagnosis of synovitis, guidance of steroid injections, and follow-up examinations of the wrist in JIA.

**Methods:**

In 11 patients (median age 12.5 years, range 2-16), 15 wrists with clinically active arthritis were assessed clinically by US and color Doppler (Logiq 9, GE, 16-4 MHz linear transducer) prior to and 1 and 4 weeks after US-guided steroid injection.

**Results:**

US detected synovitis in the radio-carpal joints, the midcarpal joints, and the tendon sheaths in 87%, 53% and 33% of the wrists, respectively. Multiple compartments were involved in 67%. US-guidance allowed accurate placement of steroid in all 21 injected compartments, with a low rate of subcutaneous atrophy. Synovial hypertrophy was normalized in 86% of the wrists, hyperemia in 91%, and clinically active arthritis in 80%.

**Conclusions:**

US enabled detection of synovial inflammation in compartments that are difficult to evaluate clinically and exact guidance of injections, and it was valuable for follow-up examinations. Normalization of synovitis was achieved in most cases, which supports the notion that US is an important tool in management of wrist involvement in JIA.

## Background

The wrist region is one of the most complex joints of the human body, and it is prone to deformity and functional impairment in juvenile idiopathic arthritis (JIA) [1,2] and is difficult to examine clinically. Inflammation in the wrist region is an indicator of poor outcome in JIA [3]. In the first detailed account of the natural history of wrist arthritis in children, Chaplin and colleagues [4] described late changes, visualized on plain radiographs, in 59% of the patients they studied. The trend towards early suppression of inflammation has probably improved the outcomes in many patients with JIA [5], but it has also increased the need for more sensitive imaging techniques.

The aim of the present study was to investigate the usefulness of ultrasonography (US) with Doppler in JIA, focusing on diagnosis of synovitis, guidance of steroid injections, and follow-up examinations of the wrist region.

## Methods

This study was conducted during 2009 and 2010 at the Department of Pediatrics, University of Copenhagen, Denmark. Patients at the Pediatric Rheumatology Outpatient Clinic, Rigshospitalet, Copenhagen, were consecutively invited to participate. The inclusion criterion was having arthritis in the wrist, which the clinician considered would benefit from intra-articular steroid therapy. Fourteen children with 18 wrists with active arthritis consented to take part. Three of those subjects were excluded after the initial US because they did not fulfill the participation requirements to show up at follow-up appointments, leaving a total of 11 children and 15 wrists for evaluation. Demographic features and the results of the clinical and laboratory assessments performed are summarized in Table 1.

**Table 1:** Clinical and laboratory assessment of 11 JIA patients with 15 symptomatic wrists*

Characteristic		Number (%)	Median	Range
Sex				

	Male	1 (9%)		

	Female	10 (91%)		

Subgroups				

	Oligoarthritis	5 (46%)		

	RF-negative polyarthritis	2 (18%)		

	Undifferentiated arthritis	2 (18%)		

	Enthesitis related arthritis	1 (9%)		

	Systemic	1 (9%)		

Age at injection, years			12.5	2-16

Disease duration, years			4.8	0.8-13.5

Number of joints (n = 15) with				

	Swelling	13 (87%)		

	Pain	14 (93%)		

	Limited range of motion	15 (100%)		

	Active arthritis	15 (100%)		

VAS wrist pain patient/parent, cm			4.0	0.2-6.0

VAS global assessment patient/parent, cm			0.5	0-3.7

VAS global assessment physician, cm			2.9	1.5-7.2

CRP level, mg/l			3	0-22

ESR, mm/h			15	4-48

HLA B27 positive, number of patients		2 (18%)		

ANA positive, number of patients		4 (36%)		

RF positive, number of patients		1 (9%)		

Drug therapies				

	MTX	5 (45%)		

	MTX + Etanercept	1 (9%)		

	MTX + Systemic corticosteroids	1 (9%)		

* ANA = antinuclear antibodies; CRP = C-reactive protein; ESR = erythrocyte sedimentation rate; HLA = human leukocyte antigen; MCTD = Mixed connective tissue disease; MTX = Methotrexate; RF = rheumatoid factor; VAS = visual analogue scale

Ten of the 11 children (91%) were female, and the median age was 12.5 years (range 2-16 years). Five had oligoarticular (oligo) JIA, two had polyarticular (poly) JIA, two had undifferentiated arthritis, one had enthesitis related arthritis (ERA) and one systemic JIA. At the time of inclusion, seven had ongoing systemic treatment: five with methotrexate, one with methotrexate and etanercept, and one with methotrexate and systemic corticosteroids (prednisolone 0.1 mg/kg). One patient had received an intra-articular steroid injection within the previous 3 months, but not in the arthritic wrist or the same extremity.

The local research ethics committee approved the study. All parents gave informed consent for their children to participate, and oral agreement was obtained from the children themselves.

### Clinical and US assessment

Patients who had previously been diagnosed with JIA based on the revised criteria of the International League of Associations for Rheumatology (ILAR, 2004) [6] were examined by either of two experienced pediatric rheumatologists for clinical signs of involvement of the wrists. The following clinical variables were recorded: swelling, pain assessed by the patient/parent visual analogue scale (VAS, 0 - 10 cm), tenderness to palpation, pain on motion, and limited range of motion.

On the same day, an initial US examination was performed on the patients with clinically active arthritis, which the ILAR defines as swelling within a joint or a limited range of joint movement with joint pain or tenderness. The US examination was conducted by a radiologist specialized in musculoskeletal US using a Logiq 9 scanner (GE Healthcare, Chalfont St. Gilles, UK) equipped with a 16-4 MHz linear transducer. The wrist of the child was imaged dorsally and palmarly with a slight palmar or dorsal flexion. B-mode US was performed to detect structural abnormalities, and color Doppler to identify hyperemia. The radio-carpal and midcarpal compartments were examined by scanning in a dorsal-longitudinal plane, and tendon sheaths (specified in Table 2) were investigated in a transversal and a longitudinal plane. The Outcome Measures in Rheumatology Clinical Trials (OMERACT) definitions for US pathology (joint effusion, synovial hypertrophy, bone erosions and tenosynovitis) in RA [7] were used, and for each of the compartments the presence/absence of these signs of pathology were registered. The findings of the color Doppler examination were assessed as presence or absence of hyperemia [8]. Color Doppler settings were standardized as follows: the pulse repetition frequency (PRF) was 0.6 KHz, the color Doppler gain was set just below the level at which noise appeared, and the wall filter was very low (65 Hz). US synovitis/tenosynovitis was defined as synovial hypertrophy with or without synovial vascularization, and with or without effusion.

**Table 2:** US diagnosis of tenosynovitis in 15 wrists before steroid injection

Tendon sheath compartments examined	Number with tenosynovitis
Extensor	

abductor pollicis longus and extensor pollicis brevis	1

extensor carpi radialis longus and brevis	2

extensor pollicis longus	1

extensor digitorum communis	3

extensor digiti minimi	3

extensor carpi ulnaris	3

Flexor	

flexor carpi radialis	3

flexor pollicis longus	2

flexor digitorum superficialis and profundus	2

**Figure 1:**
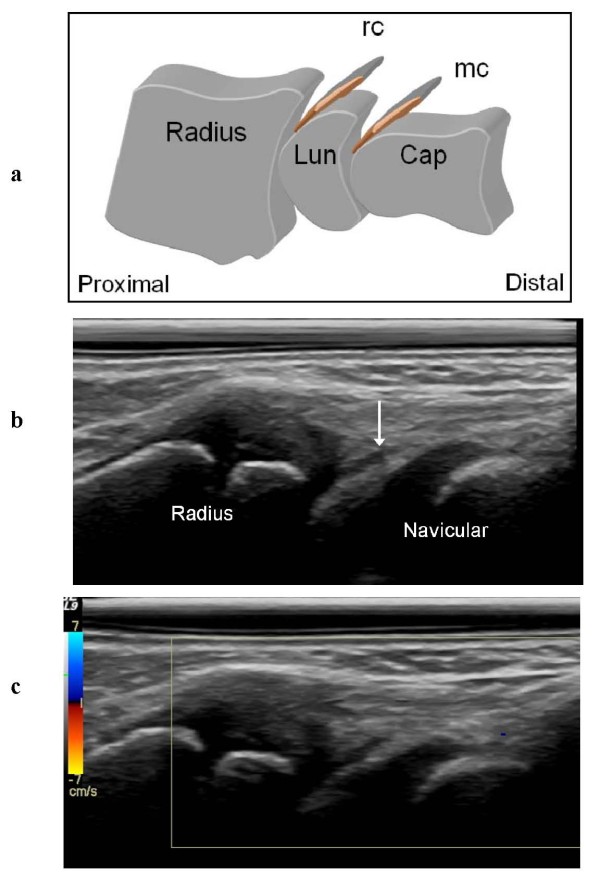
**The normal aspect of the dorsal synovial recesses of the radio-carpal and midcarpal joints**. **a **Drawing of the thin dorsal recesses of the radio-carpal (rc) and midcarpal (mc) joints, pointing distally. Lun = lunate bone; Cap = capitate bone. **b, c **Dorsal sagittal US scan showing a thin hypoechoic radio-carpal recess (**b**, arrow) without hyperemia on color Doppler examination (**c**).

**Figure 2:**
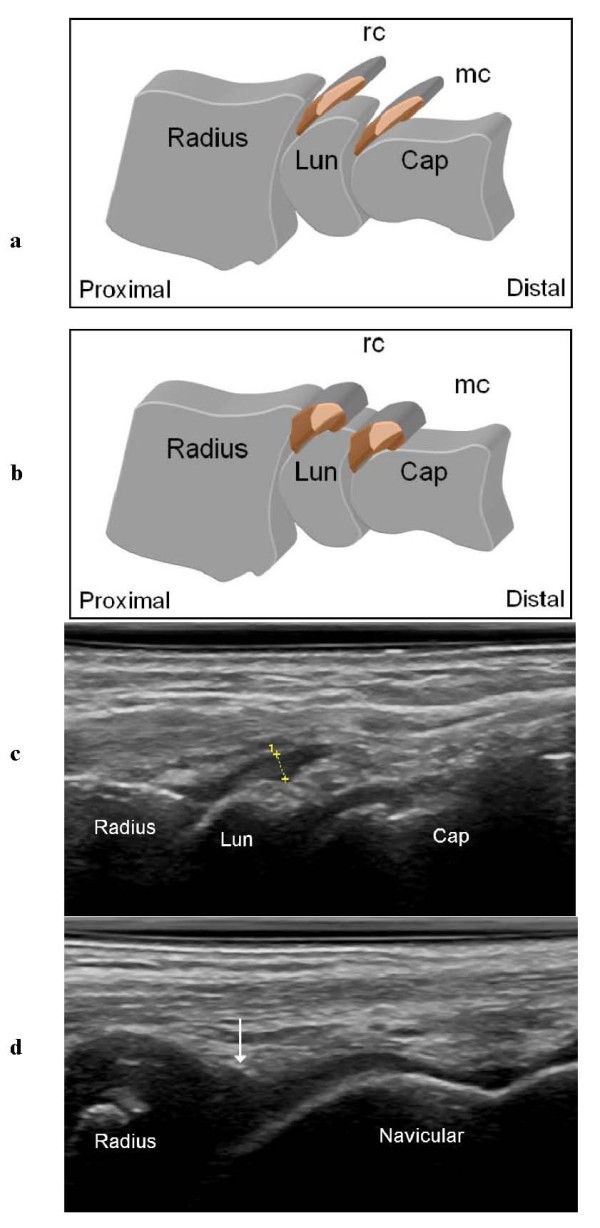
**Hypertrophic synovial recesses of the radio-carpal and midcarpal joints**. **a, b **Drawings of hypertrophic thickened (**a**) and rounded pseudotumoral (**b**) dorsal recesses of the radio-carpal (rc) and midcarpal (mc) joints. **c **Dorsal sagittal US scan of a hypoechoic thickened radio-carpal recess (1). **d **Dorsal sagittal US scan of the wrist of a 2-year-old girl in which the hypoechoic rounded radio-carpal recess (arrow) is difficult to distinguish from the hypoechoic cartilage of the radius and navicular bones. Lun = lunate bone; Cap = capitate bone.

The dorsal synovial recesses of the radio-carpal and midcarpal joints visualized by US were considered normal if they were thin (Figure 1a-c) or impossible to visualize, and as hypertrophic if they were thick or rounded (Figure 2a-d). We also registered the presence/absence of bone erosions, defined as an intraarticular discontinuity of the bone surfaces, visible in 2 perpendicular planes [7].

The results in this study are presented as absolute qualitative values without any statistical calculations due to the small size of the sample.

### US-guided steroid injection

**Figure 3:**
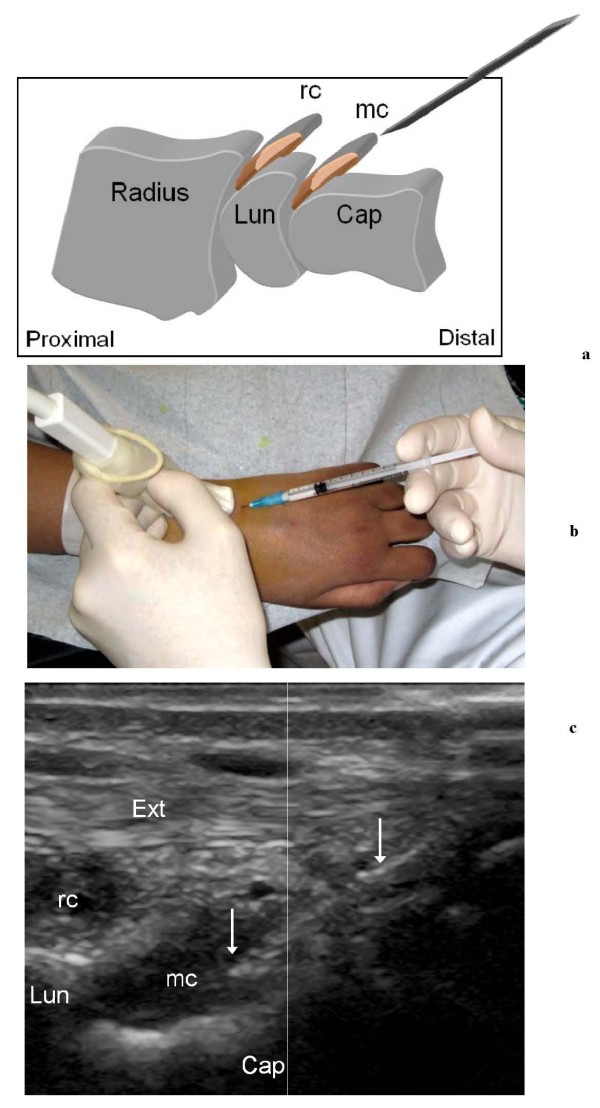
**US-guided puncture of the dorsal recess of the radio-carpal and midcarpal joints**. **a **Drawing showing the direction of the needle during puncture of the hypertrophic midcarpal recess (rc). Lun = lunate bone, Cap = capitate bone, mc = midcarpal recess. **b **Image of the US-guided puncture of the dorsal midcarpal recess in a JIA patient. **c **Dorsal sagittal US scan showing the tip of the needle (arrows) in the midcarpal recess (mc). Ext = extensor tendon, Lun = lunate bone, Cap = capitate bone, rc = radio-carpal recess.

For each patient, the clinician and the radiologist reached a consensus decision regarding whether compartments should be given injections and, if so, which ones. Injections with triamcinolone acetonide 40 mg/ml were performed in the wrist region (10-40 mg per joint and tendon sheath) [9]. US-guided injections were done by the free-hand technique using a small 12-5 MHz linear hockey-stick transducer, visualizing the position of the needle in real time. The needle (23 G) was inserted along the US plane into joint recesses or tendon sheaths. The dorsal radio-carpal and midcarpal recesses were punctured in a longitudinal scanning plane. The needle was inserted obliquely in a proximal direction in the axis of the joint recesses (Figure 3a-c), and more tangentially, controlling the needle position by an additional transverse US scan, during injection in the tendon sheaths. Aiming to minimize the risk of subcutaneous atrophy, the needle was flushed with lidocaine before withdrawal and all injections were followed by thorough local compression.

### Follow-up after injection

In all patients, the clinical and US assessment was repeated at 1 and 4 weeks after the steroid injection. The ultrasonographer performing the assessments was blinded to clinical data. For the joint recesses and tendon sheaths, the presence (no effect) or absence (normalization) of synovial hypertrophy after treatment was recorded. In the follow-up of synovial hyperemia, residual presence (no effect) or total absence (normalization) of color Doppler flow was noted.

## Results

### US results before injection

The results of Doppler US before steroid injection are summarized in Table 3. Synovial hypertrophy was found in 26 compartments and hyperemia in 23 (radio-carpal joints, midcarpal joints, and tendon sheaths). Involvement of multiple compartments was observed in 10 of the 15 wrists, and only five wrists showed isolated radio-carpal involvement. Synovitis was found in 13 (87%) of the radio-carpal joints and eight (53%) of the midcarpal joints. The compartments involved in association with the radio-carpal joint included the midcarpal joint (five wrists), tendon sheaths (two wrists), and both midcarpal joint and tendon sheaths (one wrist). In two wrists, synovial hypertrophy was detected only in the midcarpal joint and tendon sheaths. Tenosynovitis was found in five of the 15 wrists (33%), affecting a total of 5-6 tendons in three of the wrists and 1-2 tendons in two. Of all 135 tendon sheaths examined, 20 (15%) exhibited synovial hypertrophy and 16 (12%) hyperemia on Doppler-US. No patients had tenosynovitis without radio-carpal or midcarpal involvement. The age distribution was similar in patients with, and without, tendon involvement. The respective numbers of extensor and flexor tendons involved are summarized in Table 2. Color Doppler examination showed synovial hyperemia in 23 of the 26 diseased compartments (88%; Table 3). Effusion was detected in two of the 21 inflamed joint compartments and in five of the 20 diseased tendon sheaths. Bone erosions were only found in the wrist (in the os *lunatum*) of a 14 year-old girl with oligo JIA and disease duration of 13.5 years.

**Table 3:** US diagnosis of synovial hypertrophy and hyperemia in 15 wrists before steroid injection

Compartments	Synovialhypertrophy	Synovialhyperemia
Radio-carpal joints	13/15 (87%)	12/15 (80%)

Midcarpal joints	8/15 (53%)	7/15 (47%)

Tendon sheaths	5/15 (33%)	4/15 (27%)

All compartments	26/45 (58%)	23/45 (51%)

### US-guided steroid injection

US-guided steroid injection was performed in 21 of the 26 diseased compartments (summarized in Table 4). Four of the diseased tendon sheaths (two flexor and two extensor tendons) were injected. To minimize the procedural pain, injections were done under general anesthesia in younger patients (n = 8, median age 7.5 years) and with nitrous oxide-oxygen analgesia in school-age children (n = 3, median age 15 years). Nine wrists had one injection, and six wrists received two injections. The dose was 40 mg in joints and 10-20 mg in tendon sheaths (1-2 per patient). Four patients had involvement of both wrists, and both sides were injected during the same session. In five patients, other joints (three knees and two talo-crural and subtalar joints, respectively) were injected with triamcinolone hexacetonide (knees) or triamcinolone acetonide (ankle regions) during the same session. In the individual patients, the total steroid dose injected ranged from 30 to 80 mg (median 40 mg), and the US-guided steroid injection including general anesthesia took approximately 30 min (5-15 min for the steroid injection, depending on the number and sites of injections).

**Table 4:** US diagnosis of synovial hypertrophy and hyperemia 1 week and 4 weeks after US-guided injection of corticosteroids

		1 week post-injection		4 weeks post-injection	
**Compartments**	**Number injected**	**Synovial hypertrophy**	**Synovial hyperemia**	**Synovial hypertrophy**	**Synovial hyperemia**

Radio-carpal joints	12	6/12 (50%)	3/12 (25%)	2/12 (17%)	1/12 (8%)

Midcarpal joints	5	3/5 (60%)	0/5 (0%)	1/5 (20%)	1/5 (20%)

Tendon sheaths	4	0/4 (0%)	0/4 (0%)	0/4 (0%)	0/4 (0%)

All compartments	21	9/21 (43%)	3/21 (14%)	3/21 (14%)	2/21 (9%)

At the time of steroid injection, new systemic therapies were started in three of the patients: one of these children was previously untreated and was given methotrexate; one had previously received methotrexate and was switched to sulphasalazine; the third was receiving methotrexate as single therapy, and etanercept was added.

### US follow-up of steroid injection

**Figure 4:**
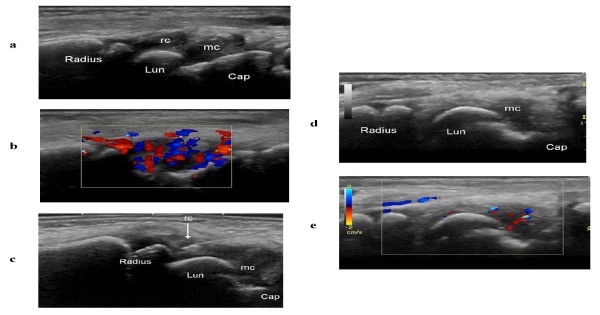
**Dorsal sagittal US scans of the radio-carpal and midcarpal recesses**. **a, b **Hypoechoic rounded radio-carpal (rc) and midcarpal (mc) recesses (**a**) with hyperemia on the color Doppler examination (**b**). Lun = lunate bone, Cap = capitate bone. **c **US-guided puncture with the tip of the needle (arrow) in the radio-carpal recess (rc). Lun = lunate bone, Cap = capitate bone, mc = midcarpal recess. **d, e **Four-week US follow-up of a midcarpal recess (mc) not treated with steroid injection, showing persistent synovial hypertrophy (**d**) and hyperemia (**e**). Lun = lunate bone, Cap = capitate bone.

US-guidance of injections enabled real-time visualization of the procedure and allowed quick and effective placement of the needle tip and steroid in all compartments. The effects on synovial hypertrophy and hyperemia in 21 injected compartments 1 and 4 weeks after treatment are shown in Table 4. After 1 week, normalization of synovial hypertrophy was noted in 57% of the injected compartments, and normalization of hyperemia was seen in 86% (Table 4); corresponding rates after 4 weeks were 86% and 91% (Table 4), respectively. After 4 weeks, Doppler-US demonstrated persistent synovitis in injected compartments in two wrists: one in a 13-year-old girl with ERA who had synovial hypertrophy without hyperemia in the radio-carpal joint; the other in a 15-year-old boy with poly JIA who showed synovial hypertrophy with hyperemia in the radio-carpal and midcarpal joints of the same wrist. The midcarpal joint was normalized on US follow-up 1 week after injection but displayed a relapse of synovial hypertrophy and hyperemia after 4 weeks. Twenty compartments (16 tendon sheaths, one radio-carpal joint, and three midcarpal joints) had US signs of disease at week 0 but were not injected. At the 4-week US follow-up, only one joint (midcarpal) exhibited residual synovial hypertrophy and hyperemia (Figure 4a-e).

At clinical follow-up 1 week after injection, eight wrists were completely free from all signs of active arthritis (joint swelling or limitation in the range of joint movement accompanied by pain or tenderness), and seven wrists still exhibited clinically active arthritis. Four weeks after injection, normalization was achieved in 12 wrists, whereas three wrists had clinically active arthritis, two in a 13-year-old girl with ERA and one in a 6-year-old girl with poly JIA.

### Relapses

A 14-year-old girl with oligo JIA was the only patient with a recurrence of symptoms in an injected wrist compartment, and this was noted 7 months after the initial steroid injection. A relapse of synovitis was verified by Doppler-US, and the wrist was re-injected with steroids under US-guidance, which led to normalization of symptoms at the clinical follow-up 1 month later.

### Complications

The only complication noted was local subcutaneous atrophy at the injected site (radio-carpal joint) in one patient, a 9-year-old girl. This represents a complication rate of 4.8%.

## Discussion

There are no validated US scoring systems for the assessment of inflammatory and joint damage abnormalities in JIA, and there is little knowledge of the normal US reference values of each joint at different developmental stages in children. In this study we used the OMERACT definitions for US pathology in adult RA [7], and the presence/absence of these signs of pathology were registered for each of the compartments examined. Recent investigations have demonstrated that clinical examination alone is inadequate to identify structures involved in JIA, and that subclinical synovitis is frequently detected by US [10,11], particularly in the hands and feet [12,13]. In the present US study, we were able to diagnose synovitis in such compartments, which are difficult to assess clinically [12,13] (Table 3).

We conducted a descriptive investigation, which was not designed to compare the results of clinical and US assessments. In symptomatic wrists, the radio-carpal and midcarpal joints were often involved (in 87% and 53%, respectively). Diseased tendon sheaths were found in only one third of the wrists, which can be compared with such findings in 55% of the symptomatic ankles examined by Doppler-US in one of our previous studies [14]. In contrast, our observation of synovial hyperemia in 88% of the diseased compartments (Table 3) is comparable to our findings in the ankle region (89%) [14] and to previous results showing hyperemia in 93% of symptomatic MCP joints [15] and 77% of symptomatic knees [16] in JIA patients. Effusion was infrequent in our patients, especially in the joint compartments.

Steroid injections constitute a major form of treatment in JIA, at disease onset or during the course of the disease [17]. The clinical effect of such an injection depends on accurate placement of the steroid in the diseased compartment. Imaging-guidance can significantly improve the accuracy, and US is the best available technique for guided injections [18-24]. Indeed, in a number of studies significantly better results were obtained using imaging-guided injections, compared to palpation-guided injections, in treatment of adults with arthritis/osteoarthritis in large and small joints [18,25] and of children with arthritis in the ankle region [26]. Common clinical practice in JIA with wrist swelling has been to perform a palpation-guided injection in the radio-carpal joint, whereas injection of the midcarpal joints or tendon sheaths is done less frequently.

To our knowledge, our study is the first to describe US-guided steroid injections in the wrist region in JIA. Inasmuch as US-guidance of injections in this region is an established practice at the Pediatric Rheumatology Outpatient Clinic, Rigshospitalet, we considered it unethical to randomize patients to either palpation-guided or US-guided injections.

Follow-up of treatment efficacy in arthritis patients is based on clinical examination and imaging. US follow-up after steroid injection or other treatments in rheumatology has been scrutinized in several studies of adults but only a few investigations of children [27-30]. Moreover, all JIA studies thus far have focused on the knee and hip. Doppler-US is widely used for follow-up in adult rheumatology, whereas the literature contains only three JIA studies in which this technique was used to evaluate treatment efficacy after steroid injections in the ankle region [14] or in knee synovitis after systemic corticosteroids [16] and NSAIDs [31]. Our study is the first to describe Doppler-US for follow-up of steroid injections in the wrist region in JIA patients. Table 4 illustrates the effect of US-guided steroid injections on synovial hypertrophy and hyperemia. In joints, normalization of hyperemia was faster than normalization of synovial hypertrophy. In tendon sheaths, complete normalization of both synovial hyperemia and hypertrophy, appeared already 1 week after steroid injection. Previous studies of JIA have shown that US and Doppler-US are more sensitive than clinical examination alone [10-13]. In the present investigation, Doppler-US revealed persistent synovitis in injected compartments in two wrists (two patients) at 4-week follow-up, and one of those wrists did not exhibit any clinical signs of active arthritis. At the same time point, results of US examination were completely normal for one of the three wrists with clinically active arthritis. Most compartments that did not receive steroid injections showed normalization 4 weeks after treatment, with the exception of one midcarpal joint. Arthrographic studies of the wrist region in children are lacking, and thus it is uncertain whether the mentioned beneficial effects were due to the existence of anatomical communications between joint compartments and tendon sheaths, or to systemic absorption of steroid. Furthermore, a number of patients received pharmacological treatments with a potential impact on the course of the disease and some received concomitant steroid injections in other joints, which might have biased the follow-up of steroid injections.

Subcutaneous atrophy is a well-recognized adverse effect of intra-articular steroid injection in pediatric patients, and it occurs most likely in small or complex joints such as the wrist or ankle in children under 4 years of age [32] or when a larger injection volume is used [33]. When employing US-guidance, as in our study, the needle tip is always correctly localized before injection, which minimizes extravasation of steroid into the subcutaneous tissue. Despite the precautions taken (see methods section for details), subcutaneous atrophy did occur in one of our patients.

**Figure 5:**
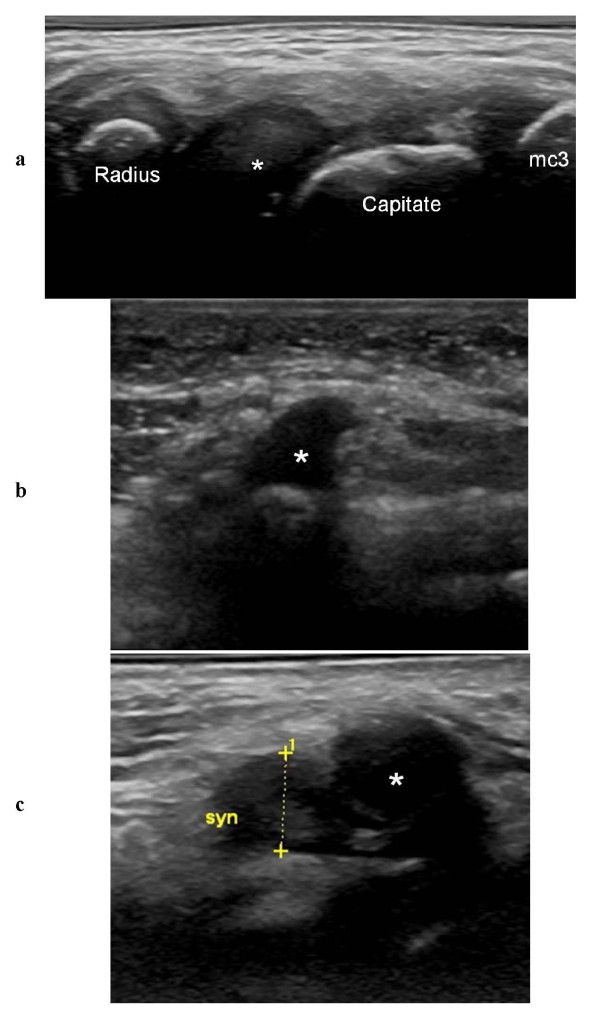
**To avoid diagnostic errors, it is important to have good knowledge of the normal US appearance of age-related ossification of cartilaginous carpal bones in young children**. **a **Dorsal sagittal US scan of the wrist in a 6-year-old girl showing a normal hypoechoic cartilaginous lunate bone (*); mc3 = metacarpal 3. **b **Palmar transversal US scan of the wrist in a 4-year-old boy showing a normal hypoechoic cartilaginous hook of the hamate (*). **c **Palmar transversal US scan of the wrist in a 2-year-old girl showing a normal hypoechoic cartilaginous pisiform bone (*) and a hypertrophic hypoechoic piso-triquetral recess (syn).

**Figure 6:**
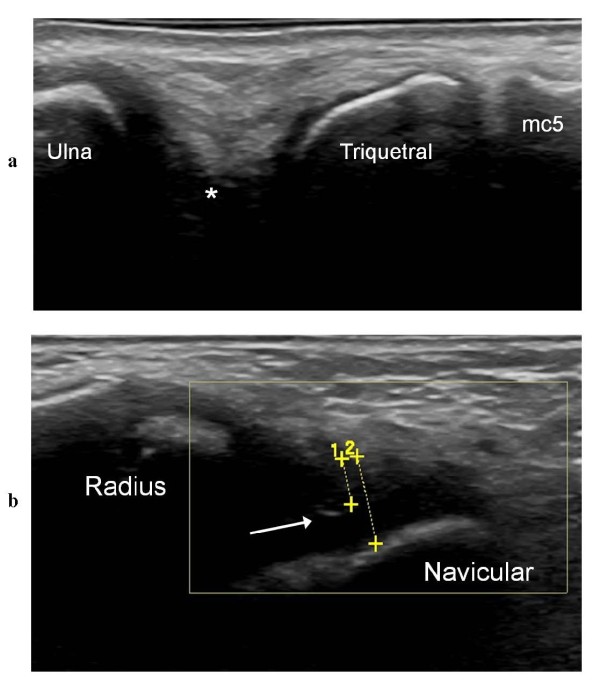
**Anisotropic artifacts in US scans of wrists of JIA patients**. **a **Dorso-ulnar longitudinal US scan of the wrist in a 14-year-old girl showing a normal large hypoechoic space (*) that was difficult to analyze due to anisotropy; mc5 = metacarpal 5. **b **Dorsal sagittal US scan showing a measurement (1) of the hypertrophic dorsal synovial recess of the radio-carpal joint. Due to anisotropy, it was difficult to differentiate between the hypoechoic synovial recess and the hypoechoic cartilage of the navicular bone. When the US beam was angled perpendicular to the surface of the cartilage, the interface between synovium and cartilage was visualized (arrow) as a small hyperechoic reflection.

US detects synovial hypertrophy as a solid, non-compressible, hypoechoic tissue in connection with joint lines or surrounding tendons [7]. Detection is more challenging in younger children than in adolescents and adults, because in the former group the synovial tissue is difficult to distinguish from the hypoechoic cartilage of the epiphyses. Therefore, to avoid diagnostic errors, it is imperative to have good knowledge of the normal age-dependent US appearance of each joint (Figure 5a-c) and also to use a meticulous scanning technique that allows clear interpretation of possible anisotropic artifacts (Figure 6a-b). The presence of juxta-articular flow at color Doppler examination in a growing child can represent either the flow of the well-vascularized cartilage of the epiphysis or synovial hyperemia reflecting disease activity.

US is suitable for examination of children of all ages and has certain advantages over MRI in that it is cheaper, mobile, quickly accessible bedside, easy to combine with clinical assessment (interactivity), and non-invasive. It does not require sedation, which facilitates repeated examinations for follow-up. Assessment of multiple locations is possible during a single session. Agitation of the patient is rarely a problem, and young children can be seated on a parent's lap or play while being examined. Modern high-frequency US transducers provide unsurpassed resolution of the superficial musculoskeletal structures in children. Doppler-US adds further information by depicting articular and para-articular soft tissue hyperemia [10,31]. The Doppler signal reflects disease activity in RA, correlating to clinical and laboratory data [34], MRI results [35], and histology [36]. Even though only a few studies have considered the use of Doppler-US for evaluation of synovial hyperemia in JIA, four investigations have demonstrated a correlation between a high synovial Doppler signal and clinical activity [8,15,16,37]. Various systems, involving quantitative or semi-quantitative methods, are used to grade the Doppler flow. In a recent US study, synovial vascularization was absent in healthy controls, and Doppler flow of any grade was significantly associated with clinical synovitis in JIA [8]. In the current study, we assessed synovial vascularization based on the presence or absence of color Doppler flow.

A weakness of the present study is that the US assessments, were evaluated for accuracy by only one experienced musculoskeletal radiologist. Furthermore, our US examination protocol did not include the radio-ulnar joint, and hence we cannot rule out any synovitis in this compartment. Accordingly, in the future we will use a revised and more appropriate scanning protocol for juvenile arthritis that includes the radio-ulnar joint.

## Conclusions

Our results highlight the value of US in pediatric rheumatology. US provided information on the exact anatomical location of inflamed structures in the wrist region, and disease was frequently found in compartments that are difficult to evaluate clinically. Also, US enabled exact guidance of steroid injections with a low rate of subcutaneous atrophy, and it was well suited for follow-up examinations. Furthermore, normalization of synovial hypertrophy and hyperemia was achieved in most cases and with few relapses, which suggests that US assessment prior to steroid injection and US-guidance of injections in this complex region have the potential to improve treatment efficacy.

## Competing interests

The authors declare that they have no competing interests.

## Authors' contributions

LL participated in design of the study, performance of clinical examinations, acquisition of data, and was responsible for analysis of the results and drafting of the manuscript. MCP was involved in design of the study, analysis of the US results, performance of US examinations and drafting of the manuscript. SN helped design the study, perform clinical examinations, and revise the manuscript. MZ contributed to design the study, the performance of clinical examinations, and revision of the manuscript. AF helped design the study, analyze the results, and draft the manuscript. All authors read and approved the final manuscript.

## References

[B1] Nieuwenhuis MK van der Net J Keessen W Kuis W Helders JM Pathokinesiology of wrist deformity in juvenile chronic arthritis: state of the art Physiotherapy Therapy and Practice 1996 12 15 25 10.3109/09593989609036414

[B2] Ansell BM Kent PA Radiological changes in Juvenile Chronic Polyarthritis Skeletal Radiol 1977 1 129 144 10.1007/BF00347138

[B3] Ravelli A Martini A Early predictors of outcome in juvenile idiopathic arthritis Clin Exp Rheumatol 2003 21 S89 93 14969057

[B4] Chaplin D Pulkki T Saarimaa A Vainio K Wrist and finger deformities in juvenile rheumatoid arthritis Acta Rheumatol Scand 1969 15 206 223 5360642 10.3109/rhe1.1969.15.issue-1-4.30

[B5] Hyrich KL Lal SD Foster HE Thornton J Adib N Baildam E Gardner-Medwin J Wedderburn LR Chieng A Davidson J Thomson W Disease activity and disability in children with juvenile idiopathic arthritis one year following presentation to paediatric rheumatology. Results from the Childhood Arthritis Prospective Study Rheumatology (Oxford) 2010 49 116 122 10.1093/rheumatology/kep352 19926670 PMC2789587

[B6] Petty RE Southwood TR Manners P Baum J Glass DN Goldenberg J He X Maldonado-Cocco J Orozco-Alcala J Prieur AM International League of Associations for Rheumatology classification of juvenile idiopathic arthritis: second revision, Edmonton, 2001 J Rheumatol 2004 31 390 392 14760812

[B7] Wakefield RJ Balint PV Szkudlarek M Filippucci E Backhaus M D'Agostino MA Sanchez EN Iagnocco A Schmidt WA Bruyn GA Musculoskeletal ultrasound including definitions for ultrasonographic pathology J Rheumatol 2005 32 2485 2487 16331793

[B8] Breton S Jousse-Joulin S Cangemi C de Parscau L Colin D Bressolette L Saraux A Devauchelle-Pensec V Comparison of Clinical and Ultrasonographic Evaluations for Peripheral Synovitis in Juvenile Idiopathic Arthritis Semin Arthritis Rheum 2011 41 272 278 10.1016/j.semarthrit.2010.12.005 21377713

[B9] Lanni S Bertamino M Consolaro A Pistorio A Magni-Manzoni S Galasso R Lattanzi B Calvo-Aranda E Martini A Ravelli A Outcome and predicting factors of single and multiple intra-articular corticosteroid injections in children with juvenile idiopathic arthritis Rheumatology (Oxford) 2011 50 1627 1634 10.1093/rheumatology/ker165 21561981

[B10] Magni-Manzoni S Epis O Ravelli A Klersy C Veisconti C Lanni S Muratore V Scire CA Rossi S Montecucco C Comparison of clinical versus ultrasound-determined synovitis in juvenile idiopathic arthritis Arthritis Rheum 2009 61 1497 1504 10.1002/art.24823 19877100

[B11] Filippou G Cantarini L Bertoldi I Picerno V Frediani B Galeazzi M Ultrasonography vs. clinical examination in children with suspected arthritis. Does it make sense to use poliarticular ultrasonographic screening? Clin Exp Rheumatol 2011 29 345 350 21385557

[B12] Haslam KE McCann LJ Wyatt S Wakefield RJ The detection of subclinical synovitis by ultrasound in oligoarticular juvenile idiopathic arthritis: a pilot study Rheumatology (Oxford) 2010 49 123 127 10.1093/rheumatology/kep339 19933594

[B13] Pascoli L Wright S McAllister C Rooney M Prospective evaluation of clinical and ultrasound findings in ankle disease in juvenile idiopathic arthritis: importance of ankle ultrasound J Rheumatol 2010 37 2409 2414 10.3899/jrheum.091262 20843904

[B14] Laurell L Court-Payen M Nielsen S Zak M Boesen M Fasth A Ultrasonography and color Doppler in juvenile idiopathic arthritis: diagnosis and follow-up of ultrasound-guided steroid injection in the ankle region. A descriptive interventional study Pediatr Rheumatol Online J 2011 9 4 10.1186/1546-0096-9-4 21276257 PMC3041992

[B15] Karmazyn B Bowyer SL Schmidt KM Ballinger SH Buckwalter K Beam TT Ying J US findings of metacarpophalangeal joints in children with idiopathic juvenile arthritis Pediatr Radiol 2007 37 475 482 10.1007/s00247-007-0438-9 17415601

[B16] Shahin AA el-Mofty SA el-Sheikh EA Hafez HA Ragab OM Power Doppler sonography in the evaluation and follow-up of knee involvement in patients with juvenile idiopathic arthritis Z Rheumatol 2001 60 148 155 10.1007/s003930170063 11475602

[B17] Bloom BJ Alario AJ Miller LC Intra-articular corticosteroid therapy for juvenile idiopathic arthritis: report of an experiential cohort and literature review Rheumatol Int 2011 31 749 756 10.1007/s00296-010-1365-x 20155422

[B18] Cunnington J Marshall N Hide G Bracewell C Isaacs J Platt P Kane D A randomized, double-blind, controlled study of ultrasound-guided corticosteroid injection into the joint of patients with inflammatory arthritis Arthritis Rheum 2010 62 1862 1869 20222114 10.1002/art.27448

[B19] Laurell L Court-Payen M Nielsen S Zak M Thomsen C Boesen M Fasth A Brasseur J, Zeitoun-Eiss D, Renoux J, Grenier P The role of ultrasonography in juvenile idiopathic arthritis (in French) In Actualités en échographie de l'appareil locomoteur 2008 Paris: Sauramps médical 73 97

[B20] Balint PV Kane D Sturrock RD Modern patient management in rheumatology: interventional musculoskeletal ultrasonography Osteoarthr Cartil 2001 9 509 511 10.1053/joca.2001.0430 11520163

[B21] Qvistgaard E Kristoffersen H Terslev L Danneskiold-Samsoe B Torp-Pedersen S Bliddal H Guidance by ultrasound of intra-articular injections in the knee and hip joints Osteoarthr Cartil 2001 9 512 517 10.1053/joca.2001.0433 11520164

[B22] Grassi W Lamanna G Farina A Cervini C Synovitis of small joints: sonographic guided diagnostic and therapeutic approach Ann Rheum Dis 1999 58 595 597 10.1136/ard.58.10.595 10491357 PMC1752775

[B23] Holm HH Skjoldbye B Interventional ultrasound Ultrasound Med Biol 1996 22 773 789 10.1016/0301-5629(96)00086-5 8923697

[B24] Christensen RA Van Sonnenberg E Casola G Wittich GR Interventional ultrasound in the musculoskeletal system Radiol Clin North Am 1988 26 145 156 3275954

[B25] Sibbitt WL Jr Peisajovich A Michael AA Park KS Sibbitt RR Band PA Bankhurst AD Does sonographic needle guidance affect the clinical outcome of intraarticular injections? J Rheumatol 2009 36 1892 1902 10.3899/jrheum.090013 19648304

[B26] Remedios D Martin K Kaplan G Mitchell R Woo P Rooney M Juvenile chronic arthritis: diagnosis and management of tibio-talar and sub-talar disease Br J Rheumatol 1997 36 1214 1217 10.1093/rheumatology/36.11.1214 9402868

[B27] Kakati P Sodhi KS Sandhu MS Singh S Katariya S Khandelwal N Clinical and ultrasound assessment of the knee in children with juvenile rheumatoid arthritis Indian J Pediatr 2007 74 831 836 10.1007/s12098-007-0148-1 17901669

[B28] Cellerini M Salti S Trapani S D'Elia G Falcini F Villari N Correlation between clinical and ultrasound assessment of the knee in children with mono-articular or pauci-articular juvenile rheumatoid arthritis Pediatr Radiol 1999 29 117 123 10.1007/s002470050554 9933332

[B29] Eich GF Halle F Hodler J Seger R Willi UV Juvenile chronic arthritis: imaging of the knees and hips before and after intraarticular steroid injection Pediatr Radiol 1994 24 558 563 10.1007/BF02012732 7724276

[B30] Sureda D Quiroga S Arnal C Boronat M Andreu J Casas L Juvenile rheumatoid arthritis of the knee: evaluation with US Radiology 1994 190 403 406 8284388 10.1148/radiology.190.2.8284388

[B31] Shanmugavel C Sodhi KS Sandhu MS Sidhu R Singh S Katariya S Khandelwal N Role of Power Doppler sonography in evaluation of therapeutic response of the knee in juvenile rheumatoid arthritis Rheumatol Int 2008 28 573 578 10.1007/s00296-007-0482-7 17987293

[B32] Job-Deslandre C Menkes CJ Complications of intra-articular injections of triamcinolone hexacetonide in chronic arthritis in children Clin Exp Rheumatol 1990 8 413 416 2397629

[B33] Beukelman T Arabshahi B Cahill AM Kaye RD Cron RQ Benefit of intraarticular corticosteroid injection under fluoroscopic guidance for subtalar arthritis in juvenile idiopathic arthritis J Rheumatol 2006 33 2330 2336 16981290

[B34] Naredo E Bonilla G Gamero F Uson J Carmona L Laffon A Assessment of inflammatory activity in rheumatoid arthritis: a comparative study of clinical evaluation with grey scale and power Doppler ultrasonography Ann Rheum Dis 2005 64 375 381 15708891 10.1136/ard.2004.023929 PMC1755396

[B35] Szkudlarek M Court-Payen M Strandberg C Klarlund M Klausen T Ostergaard M Power Doppler ultrasonography for assessment of synovitis in the metacarpophalangeal joints of patients with rheumatoid arthritis: a comparison with dynamic magnetic resonance imaging Arthritis Rheum 2001 44 2018 2023 10.1002/1529-0131(200109)44:9<2018::AID-ART350>3.0.CO;2-C 11592362

[B36] Walther M Harms H Krenn V Radke S Faehndrich TP Gohlke F Correlation of power Doppler sonography with vascularity of the synovial tissue of the knee joint in patients with osteoarthritis and rheumatoid arthritis Arthritis Rheum 2001 44 331 338 10.1002/1529-0131(200102)44:2<331::AID-ANR50>3.0.CO;2-0 11229463

[B37] Sparchez M Fodor D Miu N The role of Power Doppler ultrasonography in comparison with biological markers in the evaluation of disease activity in Juvenile Idiopathic Arthritis Med Ultrason 2010 12 97 103 21173935

